# Epidemiological and transcriptome data identify shared gene signatures and immune cell infiltration in type 2 diabetes and non-small cell lung cancer

**DOI:** 10.1186/s13098-024-01278-z

**Published:** 2024-03-12

**Authors:** Qian Yuan, Long Li, Liu-shun Wang, Shi-gui Xing

**Affiliations:** Department of Thoracic surgery, Nan Jing Gaochun people’s Hospital (The Gaochun Affiliated Hospital of Jiang Su University), 210000 Nanjing, Jiangsu China

**Keywords:** Lung cancer, Type 2 diabetes mellitus, Bioinformatics analysis, Immune cell infiltration, NHANES

## Abstract

**Background:**

Numerous previous studies have reported an association between type 2 diabetes mellitus (T2DM) and lung cancer risk, but the underlying mechanism of the interaction remains unclear. This study aimed to investigate the shared genetic features and immune infiltration processes between lung cancer and T2DM.

**Methods:**

Epidemiological data from the National Health and Nutrition Examination Survey (NHANES) 2000–2018 was used to explore the relationship between lung cancer and diabetes systematically. In addition, we also used bioinformatics methods to analyze the transcriptome data from the Gene Expression Omnibus (GEO) to explore the potential functional mechanisms from the perspective of genes and immune infiltration.

**Results:**

Logistic regression analysis showed that prediabetes (OR = 3.289,95%CI 1.231, 8.788, *p* = 0.01760, model 3)and type 2 diabetes (OR = 3.032 95%CI,1.015, 9.054, *p* = 0.04689) were significantly associated with an increased risk of lung cancer after adjusting for multiple covariates. Data from NHANES showed an inverted U-shaped relationship between fasting blood glucose and glycosylated haemoglobin and the risk of lung cancer (P for non-linear < 0.001). Transcriptome data showed that we screened 57 co-DEGs, of which 25 were up-regulated co-DEGs and 32 were down-regulated. Ten core DEGs were identified by bioinformatics analysis, which were *SMC6, CDC27, CDC7, RACGAP1, SMC4, NCF4, NCF1, NCF2, SELPLG* and *CFP*. Correlation analysis showed that some core DEGs were significantly associated with simultaneous dysregulation of immune cells.

**Conclusion:**

The identified core genes of NSCLC and T2DM are associated with dysregulated immune cells, which provides a potential research avenue for diagnosing and treating lung cancer combined with diabetes.

**Supplementary Information:**

The online version contains supplementary material available at 10.1186/s13098-024-01278-z.

## Introduction

Lung cancer is the primary cause of cancer-related death worldwide [1]. The advancement of low-dose spiral CT has led to the increased early detection of lung cancer [2]. In clinical practice, we also find that more and more patients with pulmonary nodules or lung cancer have type 2 diabetes mellitus (T2DM) and elevated blood glucose [3]. Diabetes mellitus is the most common metabolic disease and has emerged as a significant public health concern globally. According to the American Diabetes Association (ADA), diabetes is the fourth leading cause of death in the United States [4]. There exists an intricate relationship between diabetes and the incidence of various cancers.

The latest research [5] indicates that cancer patients also experience insulin resistance. Elevated insulin levels can accelerate the growth of cancer cells, thus hastening the progression of the disease. Cancers may directly affect host metabolism by secreting various factors such as tumor necrosis factor α, interleukin 6, and HIF-1, all related to metabolic regulation and/or insulin resistance. Furthermore, cancer also leads to a rewiring of fatty acid metabolism, which contributes to the onset of preclinical insulin resistance [6]. The combination of cancer and insulin resistance is highly detrimental. Identifying which cancer patients are at risk of diabetes or insulin resistance is an urgent issue that needs to be addressed.

Some studies have indicated that diabetes is linked to an increased risk of lung cancer [7]. A growing number of epidemiological and clinical studies have shown that T2DM and lung cancer (BC) co-occur in the same patient population, with a higher lung cancer risk and mortality [8]. Patients with diabetic lung cancer had significantly lower overall survival and disease-specific survival compared to non-diabetic lung cancer patients, suggesting that T2DM may be an independent prognostic factor for lung cancer [9]. While the mechanisms by which diabetes affects the advancement and treatment response of lung cancer are still unclear, increasing evidence suggests that diabetes can promote cancer through factors such as hyperglycemia, insulin resistance, hyperinsulinemia, insulin-like growth factor (IGF) expression, immune damage, and reactive oxygen species (ROS) production [10, 11]. Hyperglycemia promotes the advancement of lung cancer through high levels of insulin receptor expression, and the IGF-1/IGF-1R pathway is known to play an essential role in the pathogenesis of lung cancer [12, 13]. Additionally, patients with prediabetes have higher blood glucose levels than usual, which carries the risk of developing diabetes. There is limited research on the correlation between prediabetes and lung cancer, which warrants further exploration. Despite strong clinical and epidemiological evidence of an association between lung cancer and T2DM [12, 14, 15], the common gene regulatory mechanisms underlying diabetes and lung cancer remain unclear. The National Health and Nutrition Examination Survey (NHANES) is a research program to assess the health of adults and children in the United States [16]. Few researchers have utilized the NHANES database to investigate the association between cancer and diabetes.

This study collected the cancer incidence and diabetes status of NHANES participants from 2000 to 2018. Whether diabetes increases lung cancer risk is controversial [3, 17]. Therefore, this study aims to clarify further the positive association between diabetes and prediabetes with lung cancer using extensive sample data. Additionally, previous studies using the NHANES database mainly focused on identifying cancer risk factors through epidemiologic analyses but lacked an explanation of molecular mechanisms. The GEO (Gene Expression Omnibus) database, created by NCBI, contains high-throughput gene expression data from countries worldwide [18]. Microarray technology and integrated bioinformatics analysis are commonly used to identify novel genes and molecular mechanisms related to various diseases. According to the above reasons, we designed the present study. Initially, we analyzed a large US population cohort from 2000 to 2018 using the NHANES database, confirming the increased risk of lung cancer associated with T2DM and prediabetes. Subsequently, bioinformatics analysis was conducted based on the GEO clinical public database to explore the gene co-expression of T2DM and NSCLC and to preliminarily investigate their common pathogenic mechanism from the perspective of genes and immune infiltration. There are few studies on the mechanism of NSCLC complicated with T2DM. Exploring the common pathogenic mechanism of T2DM and NSCLC at the gene level has yet to be reported at home and abroad, which has a certain degree of innovation. This study aims to explore new gene targets and biomarkers for early lung cancer diagnosis. Genetic screening for diabetic patients with high-risk factors of lung cancer may be of great significance for early diagnosis of lung cancer. Meanwhile, we also strive to find specific gene targets for lung cancer patients with T2DM to provide new strategies for precise treatment of lung cancer patients with T2DM.

## Materials and methods

### Data sources

#### Subjects of epidemiological investigation

The epidemiological survey data in this study were collected from the NHANES dataset for nine cycles from 2000 to 2018 (https://wwwn.cdc.gov/nchs/data/nhanes/2017-2018/, accessed on 1 June 2023). The NHANES database, a significant National Center for Health Statistics (NCHS) project, includes demographic information, physical examinations, laboratory tests, and questionnaires to determine significant disease prevalence and risk factors [19, 20]. A total of 91,351 subjects were included. According to the purpose of the study, the exclusion criteria were as follows:(1) Age less than 20 years old (*n* = 41,150). (2) Questionnaire survey, fasting blood glucose, glycosylated haemoglobin, fasting insulin, and other diabetes information were missing (*n* = 29,525). (3) Cancers other than lung cancer (*n* = 1954). (4) Other demographic measures were missing (*n* = 54). Participants in the lung cancer group were identified based on the following questionnaire: “Have you ever been informed by a doctor or community health worker that you have lung cancer?” or “Have you been informed that you have any type of cancer? “. 19,543 participants were finally included in this study.

#### Gene expression data

In this study, “lung cancer”, “non-small cell lung cancer”, and “type 2 diabetes mellitus” were searched in the GEO database (https://www.ncbi.nlm.nih.gov/geoprofiles/, accessed on 13 October 2023) to obtain gene expression data of patients with lung cancer and T2DM. The selection criteria of the microarray data set were as follows: (1) It met the diagnostic criteria of lung cancer and T2DM. One data set included the diseased and the normal control groups; (2) Age > 18 years old; (3) The study data were total RNA of blood or tissue; (4) The validation sets were from the same platform, and the gender and race of the subjects were consistent. (5) All samples extracted in the data set were total RNA; (6) Patients with other cancers were excluded.

Finally, GSE18842, GSE118370, GSE26168 and GSE15932 were selected as the target data sets. GSE18842 had 47 lung cancer patients and 44 controls, and GSE118370 had 6 lung cancer patients and 6 controls. There were 18 T2DM patients and 18 controls in GSE26168, and 8 T2DM patients and 8 controls in GSE19532. Due to the small sample size of some chips, we performed a joint multi-chip analysis for the same disease. In addition, since individual chips came from different platforms, batch correction and sample normalization were performed on all datasets to eliminate individual differences among different samples. In this study, GSE135304 and GSE7014 were selected for verification. The verification set GSE135304 was derived from blood, including 200 cases and 200 normal controls. The validation set GSE7014 was derived from skeletal muscle tissue, including 6 case groups and 20 normal control groups. We substituted the obtained co-expressed genes into validation datasets to verify whether these co-expressed genes were expressed in tissues. These datasets come from a public database and do not require ethical approval. Table S1 lists all the details of these datasets.

### Diagnosis of T2DM and covariates variables

The diagnosis of diabetes was based on the question, “Have you ever been told by a doctor or health professional that you have diabetes, except during pregnancy?” Fasting blood glucose, 2 h-OGTT, and glycosylated haemoglobin were collected from all participants for diagnosis of diabetes according to the Diabetes Association (ADA) standard. The diagnostic criteria were as follows [21]: FPG ≥ 126 mg/dL (7.0 mmol/L) or 2-h plasma glucose after the OGTT test (2 h-PG) ≥ 200 mg/ dL (11.1 mmol/L) or A1C ≥ 6.5%. The diagnostic criteria for prediabetes were as follows [20]: FPG ≥ 100 mg/dL (5.6 mmol/L) and ≤ 125 mg/dL (6.9 mmol/L) or a 2 h-PG ≥ 140 mg/ dL (7.8 mmol/L) and ≤ 199 mg/dL (11.0 mmol/L) or an HbA1c ≥ 5.7% and ≤ 6.4%.

Covariates associated with diabetes or lung cancer were collected from the NHANES database by previous studies. The covariates included age (year), gender, race (Non-Hispanic White, Non-Hispanic Black, Mexican American, Others), BMI (kg/m2), Educational level (less than high school, high school, more than high school), Hypertension history, total cholesterol (TC), triglyceride (TG), lower density lipoprotein (LDL), higher density lipoprotein (HDL), smoking status and alcohol consumption.

### Weighted gene co-expression network analysis (WGCNA)

WGCNA is a commonly used method to construct a gene co-expression network and explore the association between the phenotype of interest and the core genes in the network. Moreover, enrichment analysis was conducted for each module gene. The WGCNA analysis consisted of two main parts: expression clustering analysis and phenotypic association analysis, including gene co-expression network construction, module identification, module information extraction, module and trait association, and regulatory relationship of genes within modules. Initially, we obtained the expression profiles of the NSCLC and T2DM datasets after batch correction for further analysis. We utilized the WGCNA package (version 1.69) in R (version 4.2.1, R Foundation for Statistical Computing, Vienna, Austria) to construct a co-expression network for differentially expressed genes. We constructed a weighted adjacency matrix with weighted correlation coefficients and transformed the adjacency matrix into a matched overlap matrix (TOM). Subsequently, the hierarchical clustering method was used for module identification and feature gene calculation. Finally, gene significance and module membership were calculated to correlate with clinical features. Genes involved in the corresponding modules were used for subsequent analyses. Finally, the feature gene network was visualized.

### Identification of differentially expressed genes (DEGs)

The limma package (version 3.5.1) on the R platform was used to identify DEGs between NSCLC and T2DM. Genes with logFC > logFold Change and adj.*p*-value < adjust *p* were considered up-regulated, while genes with logFC<(-logFold Change)and adj.*p*-value < adjust*p* were considered down-regulated. Finally, the DEGs of lung cancer and T2DM were intersected (only the genes that were both up-regulated or down-regulated) to obtain co-expressed genes (co-DEGs), visualized using the Venn Diagram package (version 1.7.3). Statistical difference was defined as *p*-value < 0.05.

### Enrichment analyses

Enrichment analysis was used to explore the functions of genes and more comprehensive biological information. GO enrichment analysis and Kyoto Encyclopaedia of Genes and Genomes (KEGG) pathway enrichment were performed using the Cluster Profiler package (version 4.2.2) on the R platform. The results of GO enrichment analysis were categorized into three functional categories: Biological Process (BP), Cell Component (CC) and Molecular Function (MF). Visualization of the results was done using bar and dot plots. Statistical differences were defined as *p* < 0.05.

### Construction of PPI network and screening of key genes

PPI networks for NSCLC, T2DM and co-DEGs were established using the online tool STRING 26 (http://string-db.org). We set the interaction association to the maximum confidence score of > 0.4 and hide the disconnected nodes in the network. To filter out co-DEGs with weak associations and disconnected nodes, we set the low confidence score of co-DEGs > 0.150. In the end, for a better understanding of the protein-protein interaction and to select the function of the gene interaction, we used Cytoscape (version 3.9.1) software (http://www.cytoscape.org/) for the visualization and analysis of the PPI network. The genes in the PPI network were ranked, and the top 10 genes identified as hub genes in each algorithm were selected. Finally, the critical hub genes co-expressed in NSCLC and T2DM were determined.

### Diagnostic efficacy of hub genes

Receiver Operating Characteristic Curve Analysis (ROC) was utilized to assess the diagnostic value of a specific factor for diagnosing a particular disease. ROC plots depict the relationship between sensitivity and specificity. The area under the curve (AUC) represents the accuracy of the detection method, with a value ranging from 0.5 to 1. The closer the AUC is to 1.0, the higher the authenticity of the detection method. We evaluated the accuracy of hub gene prediction and used ROC analysis to differentiate lung cancer groups from normal controls and T2DM from normal controls. The ROC curve of hub genes was generated using the pROC package (version 1.18.0) on the R platform, and the area under the ROC curve and AUC were measured to compare the diagnostic value of hub genes.

### Immune infiltration analyses

We performed immune infiltration assays using CIBERSORT. CIBERSORT [22] is a deconvolution algorithm used to analyze gene expression data and uses gene expression tags to determine the proportion of each immune cell type. The R script was downloaded from the CIBERSORT website (https://cibersortx.stanford.edu/). We used the original CIBERSORT gene signature file LM22 (LM22 defines 22 immune cell subtypes) to assess the infiltration status of 22 immune cells in the lung cancer dataset. We then used the “ggplot2” package (version 3.4.4) to draw boxplots to visualize the differences in immune cell infiltration in the lung cancer group compared with the control group. Finally, TIMER, a web server for comprehensive analysis of tumor-infiltrating immune cells (https://cistrome.shinyapps.io/timer/), was used to explore the association between immune cells and the identified core genes.

### Statistical methods

**Fig. 1:**
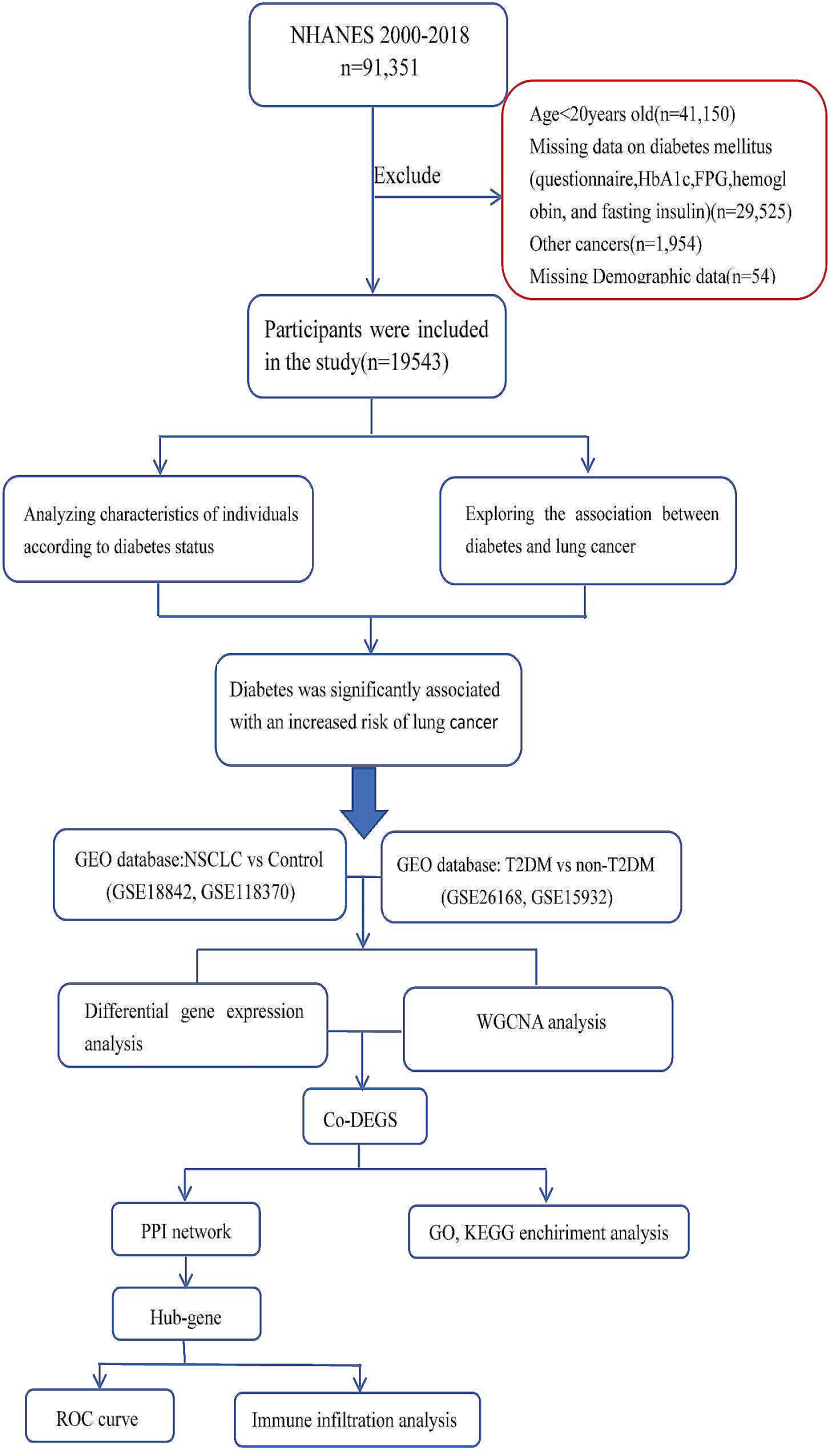
Flowchart of this study

R (version 4.2.1, R Foundation for Statistical Computing, Vienna, Austria) and Empower Stats (version 2.0, Boston, Massachusetts, USA) were used for all statistical analyses. The Kruskal-Wallis rank sum test was used for continuous variables, and the chi-square test with design adjustment was used for categorical variables. Fisher’s exact probability test was used if the count variable had a theoretical number < 10. Population characteristics were reported as means ± standard deviations for continuous variables and N (percentages) for categorical variables. A multivariate logistic regression model was used to analyze the association between diabetes status and the risk of lung cancer. Three models were used for the analysis: Model 1 did not account for confounding variables, and Model 2 adjusted for age, sex, and race. Based on Model 2, Model 3 also considered the influence of BMI, education, TC, TG, HDL, LDL, drinking history, smoking status, and history of hypertension. Odds ratios and 95% confidence intervals (CI) were used to describe the results. Restricted cubic splines show nonlinear relationships between lung cancer risk and glycemic measures. *P* < 0.05 was considered statistically significant. The flow chart is presented in Fig. 1.

**Table 1:** Weighed baseline characteristics of all participants

Characteristic	Type 2 diabetes	Prediabetes	Non-diabetes	**P**-value
Number of subjects (n)	8828	7943	2771	
Age (years)	40.835 ± 16.297	52.513 ± 16.633	60.028 ± 13.727	< 0.001
Gender (%)				< 0.001
Male	3645 (41.289)	4344 (54.690)	1518 (54.782)	
Female	5183 (58.711)	3599 (45.310)	1253 (45.218)	
Race (%)				< 0.001
Non-Hispanic White	4103 (46.477)	3260 (41.042)	917 (33.093)	
Non-Hispanic Black	1683 (19.064)	1647 (20.735)	686 (24.756)	
Mexican American	1476 (16.720)	1428 (17.978)	595 (21.472)	
Others	1566 (17.739)	1608 (20.244)	573 (20.678)	
BMI (kg/m2)	27.226 ± 6.011	29.834 ± 6.845	32.056 ± 7.370	< 0.001
Glycohemoglobin (%)	5.203 ± 0.285	5.635 ± 0.369	7.616 ± 1.841	< 0.001
Fasting plasma glucose (mmol/L)	5.068 ± 0.394	5.857 ± 0.479	9.442 ± 3.602	< 0.001
Fasting insulin (uU/mL)	9.849 ± 8.299	14.499 ± 14.152	23.492 ± 31.831	< 0.001
Total cholesterol (mmol/L)	4.982 ± 1.074	5.126 ± 1.068	4.906 ± 1.228	< 0.001
Higher density lipoprotein (mmol/L)	1.474 ± 0.434	1.354 ± 0.397	1.255 ± 0.373	< 0.001
Lower density lipoprotein (mmol/L)	2.919 ± 0.891	3.082 ± 0.908	2.798 ± 0.953	< 0.001
Triglyceride (mmol/L)	1.301 ± 1.031	1.537 ± 1.184	1.994 ± 2.162	< 0.001
Educational level (%)				< 0.001
Less than high school	1919 (21.738)	2271 (28.591)	1033 (37.279)	
High school	1898 (21.500)	1931 (24.311)	647 (23.349)	
More than high school	5011 (56.763)	3741 (47.098)	1091 (39.372)	
Alcohol (%)				< 0.001
No	2213 (25.068)	2112 (26.589)	940 (33.923)	
Yes	5803 (65.734)	5105 (64.270)	1573 (56.767)	
Not reported	812 (9.198)	726 (9.140)	258 (9.311)	
Smoking (%)				
No	5232 (59.266)	4141 (52.134)	1352 (48.791)	
Yes	3596 (40.734)	3802 (47.866)	1419 (51.209)	
Hypertension history (%)				< 0.001
No	7067 (80.052)	4834 (60.859)	1070 (38.614)	
Yes	1761 (19.948)	3109 (39.141)	1701 (61.386)	
Lung cancer (%)				< 0.001
No	8823 (99.943)	7916 (99.660)	2760 (99.603)	
Yes	5 (0.057)	27 (0.340)	11 (0.397)	

Continuous variables: mean ± standard deviation; Categorical variables: number (%)

## Results

### The baseline characteristics of individuals in 2000–2018 NHANES

The flow chart of this study is shown in Fig. 1. The patients were grouped according to diabetes status, and the differences in clinical characteristics among the non-diabetes group, pre-diabetes group and type 2 diabetes group were compared. The results showed that gender, age, race, education level, BMI, TC, TG, HDL, LDL, FPG, Fins, Hba1c, drinking history, smoking status, and history of hypertension significantly differed among the three groups (Table 1). Although the incidence of lung cancer was low in the study cohort, the incidence of lung cancer gradually increased among the three groups.

**Fig. 2:**
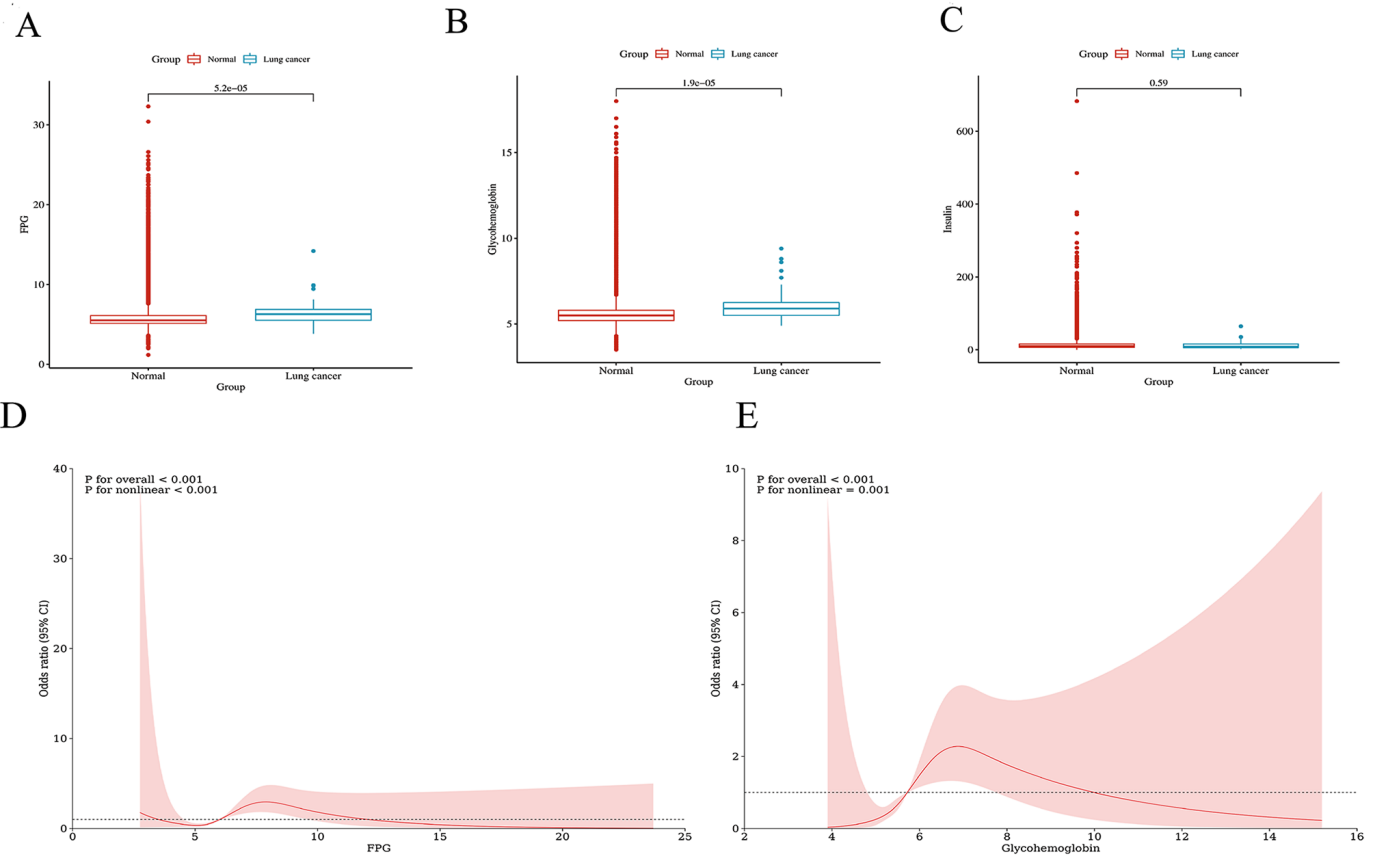
The Association between diabetes and lung cancer. (**A**-**C**) FPG, glycosylated haemoglobin, and fasting insulin in the lung cancer group and control group; (**D**) The association between FPG and the risk of lung cancer; (**E**) The association between glycosylated haemoglobin and the risk of lung cancer

The results of whether or not lung cancer occurred are shown in Fig. 2; FPG and glycosylated haemoglobin were significantly different between the two groups. FPG and glycated haemoglobin levels (Fig. 2A and B) were higher among participants with lung cancer. Although the fasting insulin level was lower in the lung cancer group than in the non-lung cancer group, the difference between the two groups was insignificant (Fig. 2C).

### The association between diabetes status and lung cancer

Table 2 shows the association between diabetic status (non-diabetic, pre-diabetic, and type 2 diabetes) and lung cancer using multiple logistic regression analysis. The results were presented with odds ratios (ORs) and 95% confidence intervals (CIs) of the three different models. The ORs of all three models were considered the reference group for the non-diabetic group. For the pre-diabetes group, the OR value of model 1 was 6.019 (95%CI 2.317, 15.637, *p* = 0.00023), and the OR value of model 2 was 3.334 (95%CI 1.259, 8.829, *p* = 0.01540). The OR value of model 3 was 3.289 (95%CI 1.231, 8.788, *p* = 0.01760). For the type 2 diabetes group, the OR of model 1 was 7.033 (95%CI 2.441, 20.259, *p* = 0.00030), and the OR of model 2 was 3.032 (95%CI 1.015, 9.054, *p* = 0.04689). The OR of model 3 was 3.110 (95%CI 0.999, 9.684, *p* = 0.05020).

**Table 2:** Relationship between diabetes status and lung cancer

	Model I	Model II	Model III
	OR (95% CI, *P*)	OR (95% CI, *P*)	OR (95% CI, *P*)
Non-diabetes	Reference	Reference	Reference
Prediabetes	6.019 (2.317, 15.637) 0.00023	3.334 (1.259, 8.829) 0.01540	3.289 (1.231, 8.788) 0.01760
Type 2 diabetes	7.033 (2.441, 20.259) 0.00030	3.032 (1.015, 9.054) 0.04689	3.110 (0.999, 9.684) 0.05020

Model I: No covariates were adjusted; Model II: Adjusted for sex, age, race; Model III: Adjusted for sex, age, race, BMI, education, TC, TG, HDL, LDL, drinking history, smoking status, and history of hypertension

These findings suggest that T2DM is significantly associated with increased risk for lung cancer, even after adjustment for multiple covariates. The results are detailed in Table 2.

In U.S. adults, fasting blood glucose and glycosylated haemoglobin were nonlinearly associated with lung cancer risk. As shown in Fig. 2, fasting blood glucose and glycosylated haemoglobin had an inverted U-shaped relationship with the risk of lung cancer (*P* for non-linear < 0.001). In summary, diabetic status and glycemic measures were significantly associated with an increased risk of lung cancer.

### Identification of differentially expressed genes (DEGs)

**Fig. 3:**
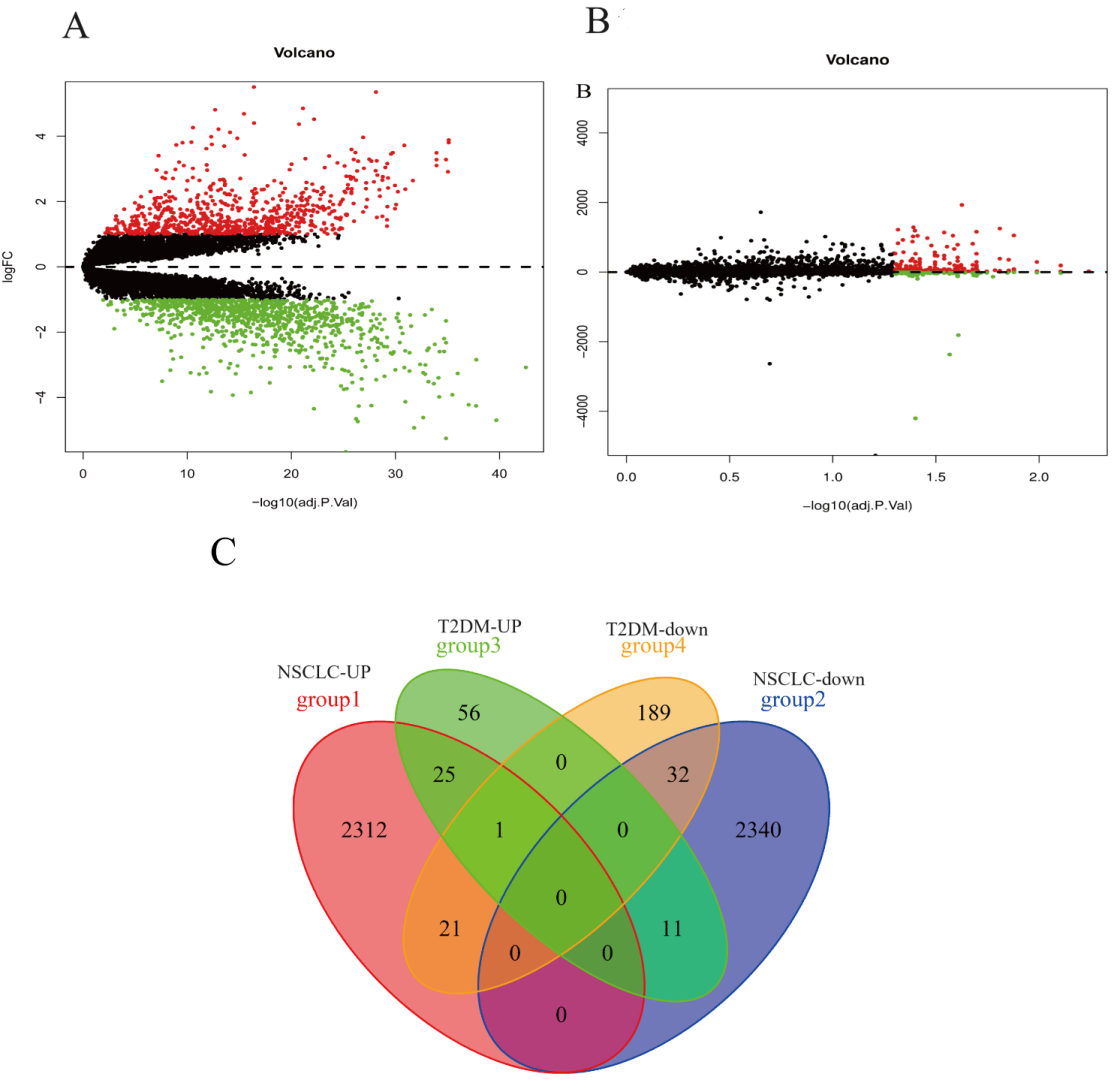
volcano maps of (**A**) Lung cancer and (**B**) T2DM;(**C**) Venn diagram of co-DEGs

This study analyzed datasets obtained from the GEO database using R language. The lung cancer group was compared with the control group, and 4741 DEGs were found, including 2358 up-regulated genes and 2383 down-regulated genes. A total of 334 DEGs (including 92 up-regulated genes and 242 down-regulated genes) were identified between T2DM patients and normal controls. Subsequently, the intersection of NSCLC-DEGs and T2DM-DEGs was taken on the R platform to analyze their co-DEGs, and the results were visualized using a Venn diagram (Fig. 3C). We identified 57 co-DEGs, including 25 up-regulated co-DEGs (*RACGAP1, MYBL2, ASUN, SMC4, HN1L, BRI3BP, ORC5, UNG, SMC6, ALG6, DROSHA, GOLT1B, FAM69A, G2E3, ABCE1, NCAPD3, RNMT, CDC27, YWHAG, SLC5A3, DLG1, POT1, CDC7, PIGW, ZNF322*) and 32 down-regulated co-DEGs(*NXB, GSTM5, RILPL2, PREX1, HSPB8, AGTR1, ARRB2, NCF2, FAXDC2, SELPLG, ALDH2, NCF1C, CFP, NCF4, IL6R, ARID5A, NCF1, IL16, MID1IP1, PALM, MCL1, ATP6V0D1, TFEB, FRAT1, SIGLEC5, RAB24, LSP1, PYCARD, SOD2, TREM2, CAPG, ADGRG3*). Figure 3A-B shows the volcano diagram.

### Identification of co-expression modules by WGCNA

**Fig. 4:**
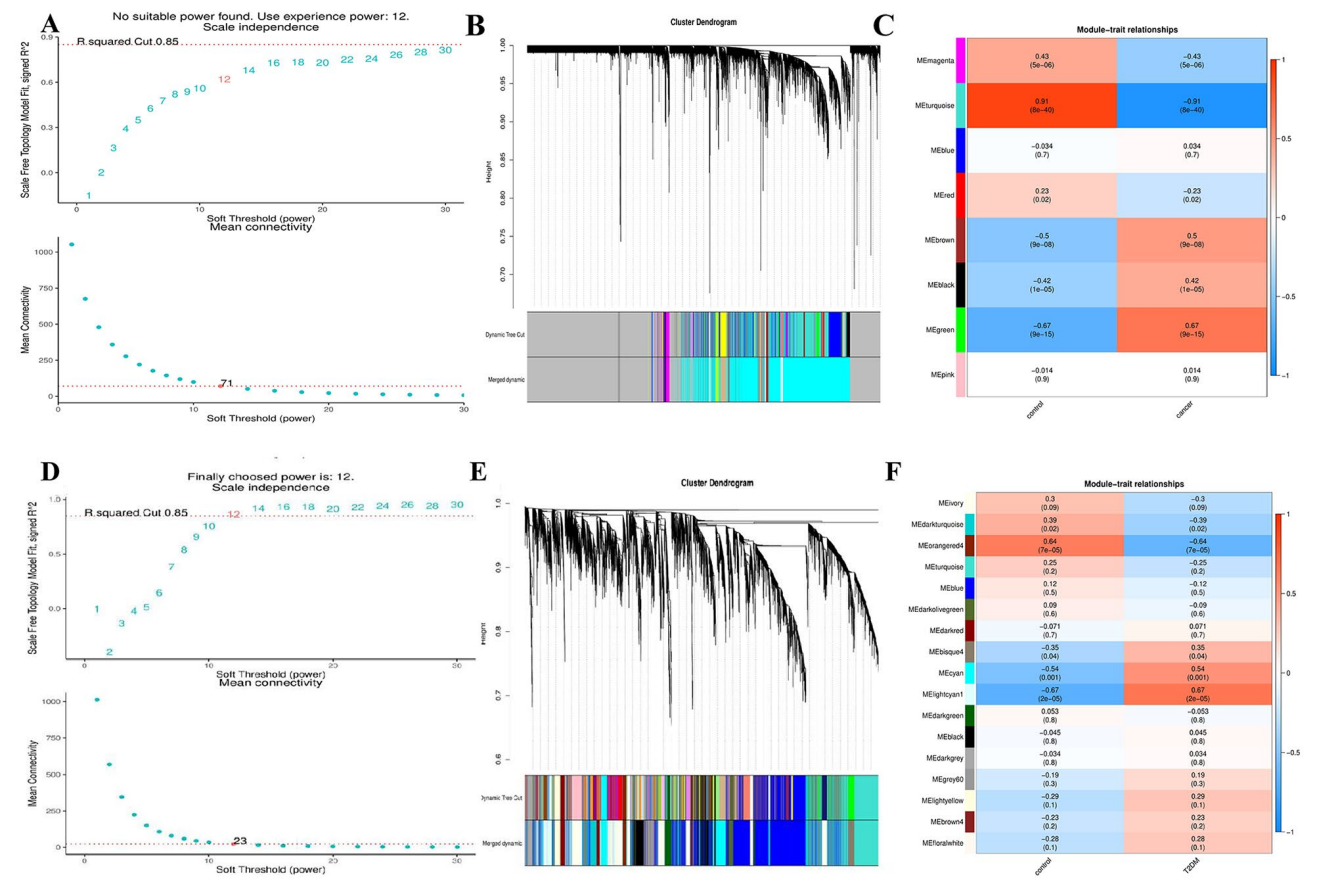
WGCNA for (**A**,**D**) the scale-free index for various soft-threshold powers (β) and the mean connectivity for various soft-threshold powers for NSCLC and T2DM; (**B**,**E**) phyloclustering tree map of the genes for NSCLC and T2DM; (**C**,**F**) heatmap of module and trait / grouping correlation for NSCLC and T2DM

WGCNA was used to construct a co-expression module to evaluate whether there was a co-expression pattern of each gene between samples and to determine whether NSCLC genes and T2DM genes had the same expression pattern in a particular stage. By constructing a weighted gene co-expression network, we set the soft threshold to 12 to guarantee high gene independence and low average connectivity to identify co-expressed gene modules. Based on the weighted correlation, hierarchical clustering analysis was conducted, and the cluster results were determined according to the set criteria. The analysis outcomes were depicted using cluster trees with different branches and colors. In this study, we analyzed the expression matrices of all samples in the NSCLC and T2DM datasets separately. We selected variant genes in the top 30 to 50% (less than 5000) for co-expression analysis. We calculated module signature genes representing each module’s overall gene expression level clustered according to their correlation. In addition, we generated the heatmap to show the correlation between modules and a given trait or grouping, with the trait or grouping on the abscordinate and the module on the ordinate; the redder the color in the heatmap, the stronger the positive correlation. On the contrary, the bluer the color, the stronger the negative correlation. The values in the grid are the correlation coefficients and p-values, respectively. If a trait or grouping is linked to a module with an absolute value closer to one, it is likely associated with the trait or grouping’s gene function in that module. WGCNA identified eight modules in the lung cancer data, and the interrelationship between the modules was assessed. MEmagenta exhibited a highly negative correlation with NSCLC, while MEgreen showed a positive correlation with NSCLC. In addition, 17 modules were found in the diabetes data, with the MEorangered4 module displaying a negative correlation with T2DM, and the MElightcyan1 module was highly positively correlated with T2DM. We retained the genes associated with these modules for further analysis. The results of all WGCNA analyses are shown in Fig. 4.

### Functional enrichment analysis

**Fig. 5:**
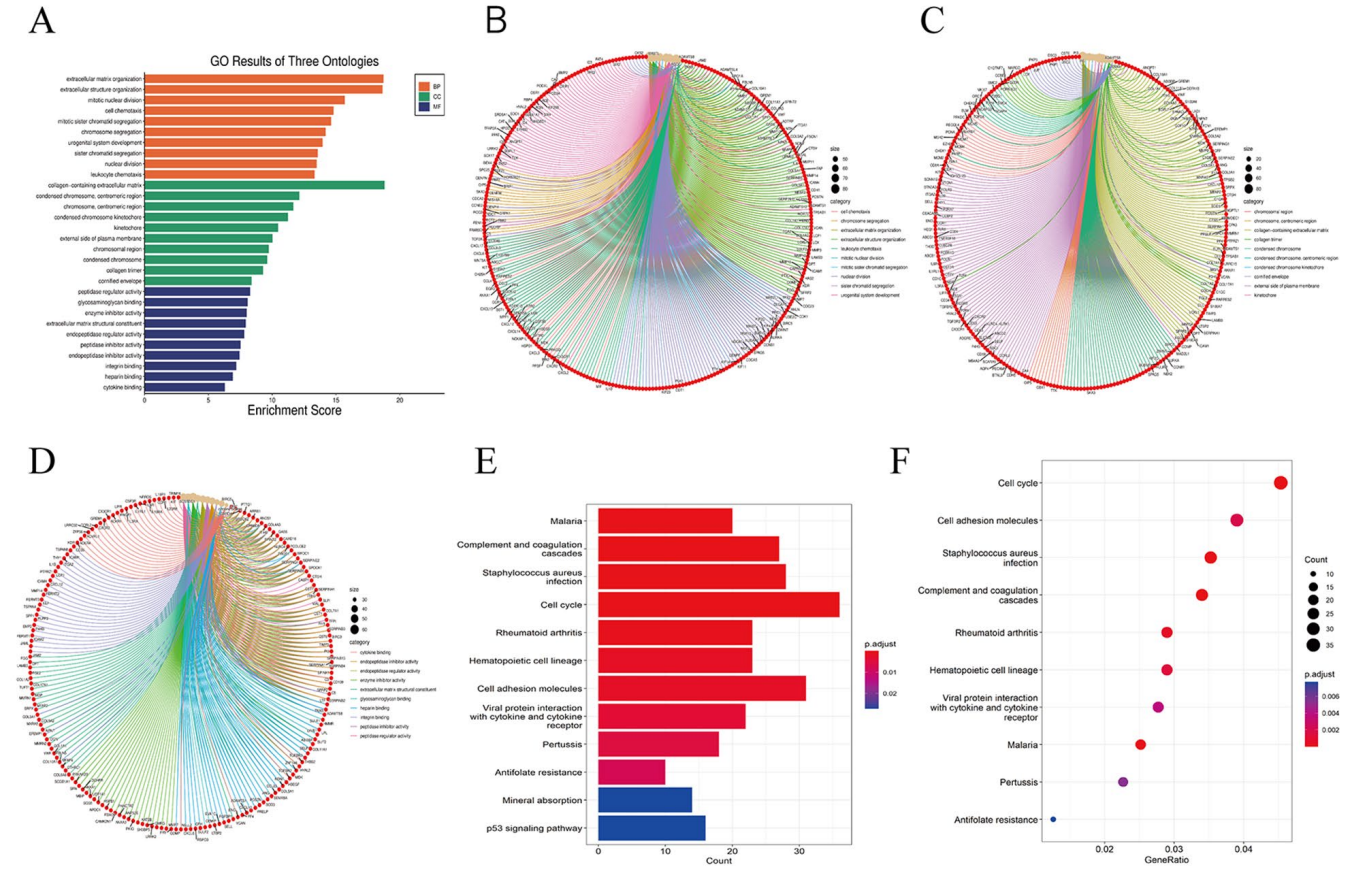
Functional characteristics analysis for NSCLC. (**A**) GO enrichment results. (**B**) Go-enriched BP; (**C**) Go-enriched CC; (**D**) GO MF; (**E**) KEGG enriched barplot; (**F**) Dot plot of KEGG enrichment

GO and KEGG enrichment are two main methods for analyzing gene function and structure. Firstly, GO and KEGG enrichment analyses were performed on lung cancer. GO enrichment analysis identified the biological process (BP), cellular component (CC), and molecular function (MF) of these genes, respectively. From the GO analysis of NSCLC (Fig. 5A), biological process (BP) (Fig. 5B) showed that DEGs were mainly enriched in an extracellular matrix organization, extracellular structure organization, extracellular matrix organization, mitotic nuclear division, cell chemotaxis, and mitotic sister chromatid segregation. The cellular component (CC) (Fig. 5C) consisted mainly of collagen-containing extracellular matrix, condensed chromosome-centromeric region, chromosome-centromeric region, and condensed chromosome kinetochore, kinetochore. Molecular functions (MF) (Fig. 5D) mainly included peptidase regulator activity, glycosaminoglycan binding, and enzyme inhibitor activity. KEGG pathway analysis (Fig. 5E-F) showed that DEGs were mainly enriched in the Cell cycle (*p* = 0.00011), Complement and coagulation cascades (*p* < 0.0001), Staphylococcus aureus infection (*p* = 0.00068), Hematopoietic cell lineage (*p* = 0.00168), Cell adhesion molecules (*p* = 0.002091), Viral protein interaction with cytokine and cytokine receptor (*p* = 0.000448), Antifolate resistance (*p* = 0.008433), and p53 signaling pathway (*p* = 0.028114).

**Fig. 6:**
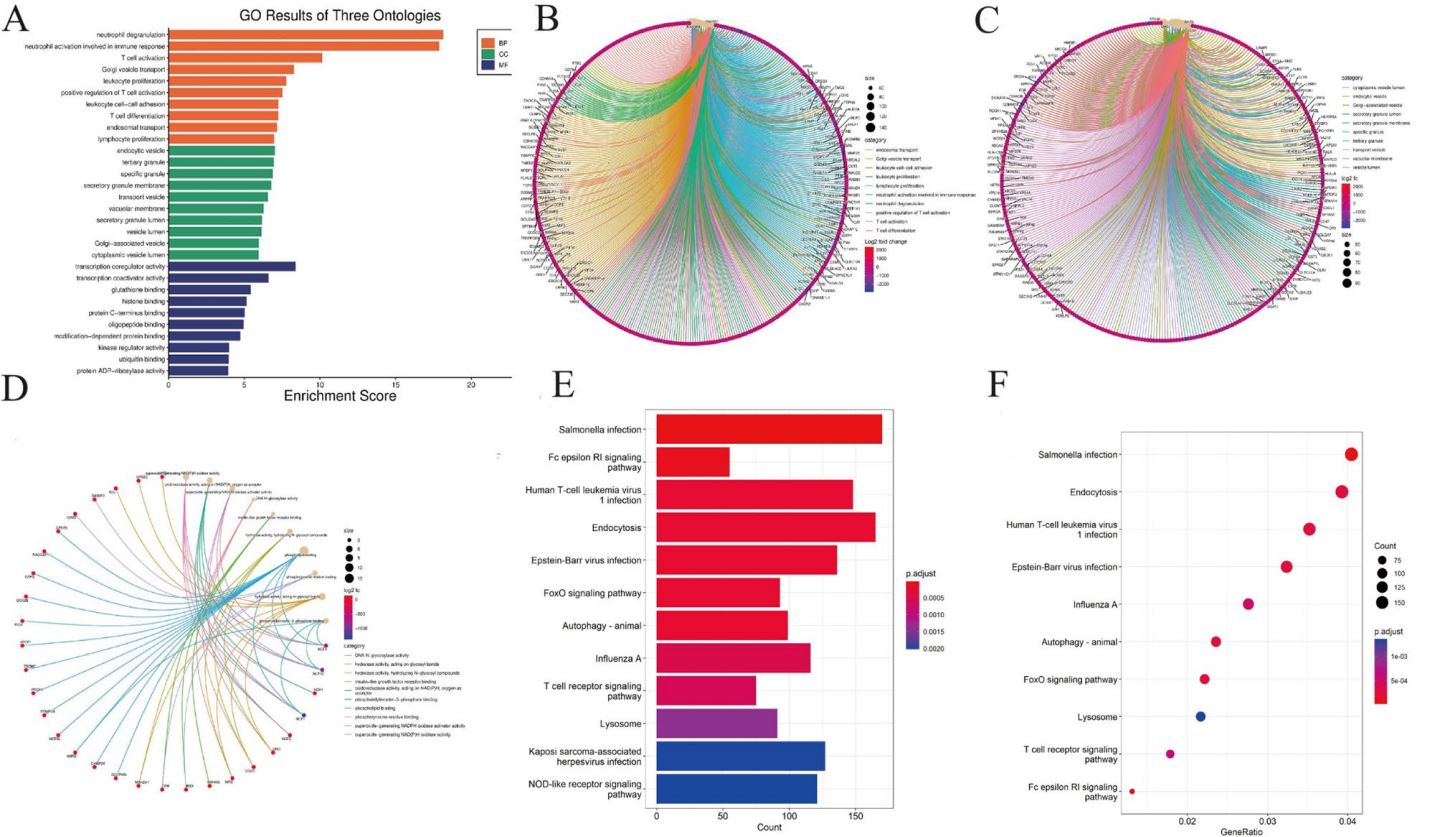
Functional characteristics analysis for the T2DM. (**A**) GO enrichment results. (**B**) Go-enriched BP; (**C**) Go-enriched CC; (**D**) GO MF; (**E**) KEGG enriched barplot; (**F**) Dot plot of KEGG enrichment

GO analysis of T2DM-DEGs is shown in Fig. 6A. Biological processes (BP) (Fig. 6B) showed that DEGs were mainly enriched in the regulation of intrinsic apoptotic signaling pathways, regulation of apoptotic signaling pathways, and superoxide metabolism. The cellular component (CC) (Fig. 6C) consists mainly of NADPH oxidase complexes, Fleming bodies, endocytic vesicles, and secondary lysosomes. Molecular function (MF) (Fig. 6D) mainly includes NADH oxidase activity for superoxide production, oxidoreductase activity, NADPH oxidase activator activity for superoxide production, and DNA-glycosylase activity. KEGG pathway analysis (Fig. 6E-F) was mainly enriched in several metabolic diseases, such as Lipid and atherosclerosis (*p* = 0.003051), fat digestion and absorption (*p* < 0.0001), and some signaling pathways, such as neurotrophic factor signaling pathway (*p* = 0.022672)and AGE-RAGE signaling pathway (*p* < 0.0001).

**Fig. 7:**
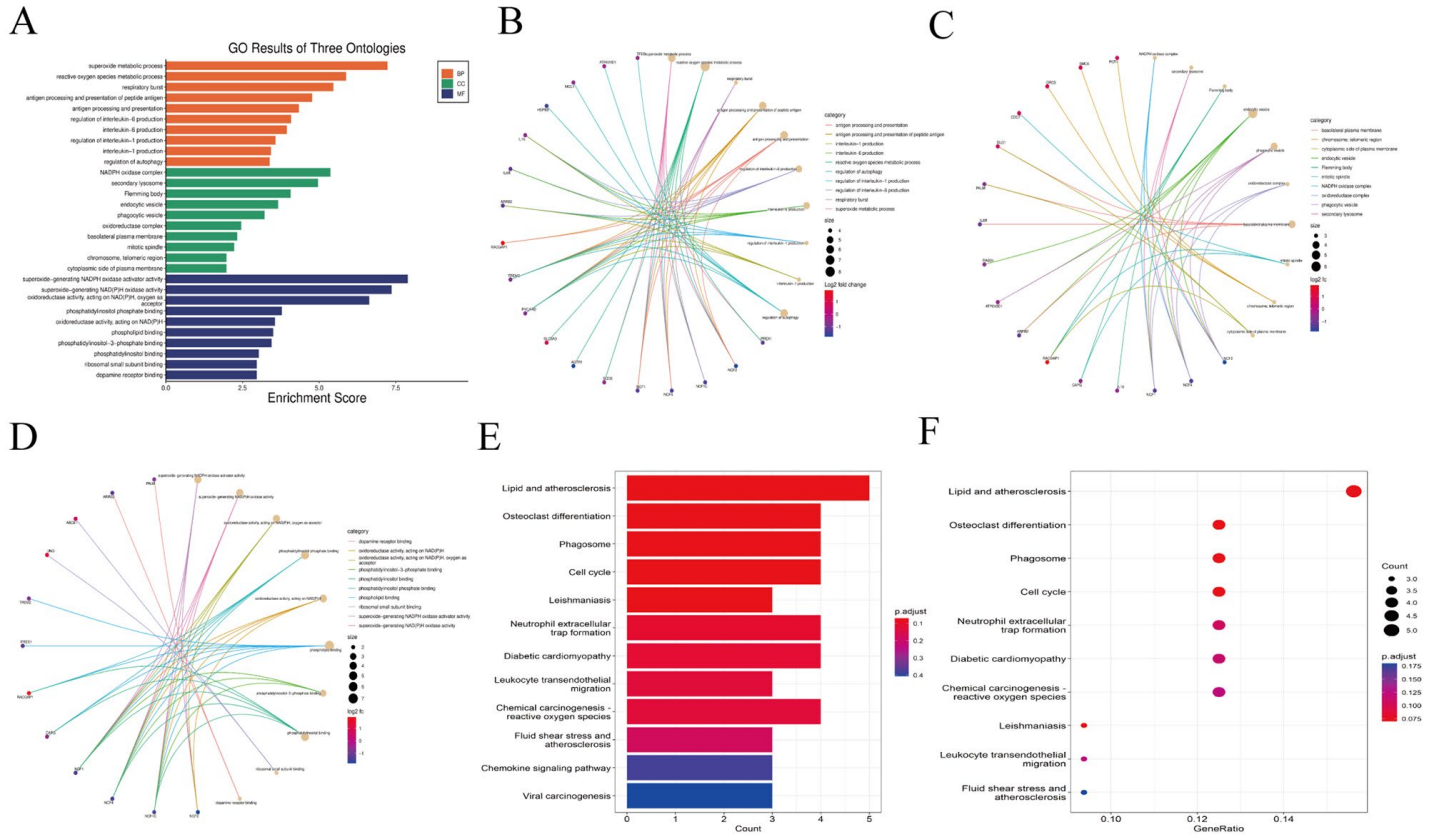
Functional characteristics analysis for the co-DEGs. (**A**) GO enrichment results. (**B**) Go-enriched BP; (**C**) Go-enriched CC; (**D**) GO MF; (**E**) KEGG enriched barplot; (**F**) Dot plot of KEGG enrichment

In addition, GO functional enrichment analysis and KEGG enrichment analysis were performed to explore co-DEGs’ biological functions further. GO pathway analysis of co-DEGs (Fig. 7A) showed that Changes in biological processes (BP) (Fig. 7B) mainly include the superoxide metabolic process, reactive oxygen species metabolic process, and reactive oxygen species metabolic process. The cellular component (CC) (Fig. 7C) was mainly enriched in NADPH oxidase complex, secondary lysosome, and Flemming body. In terms of molecular function (MF) (Fig. 7D), co-DEGs were mainly enriched in superoxide generating NADPH oxidase activator activity, superoxide generating NAD(P)H oxidase activity oxidoreductase activity, acting on NAD(P)H, and oxygen as acceptor. As for the enrichment of KEGG analysis (Fig. 7E-F), the result of the co-DEGs mainly enriched in some inflammation and metabolic diseases, such as Lipid and atherosclerosis (*p* = 0.001190)), Neutrophil extracellular trap formation (*p* = 0.005241), Diabetic cardiomyopathy (*p* = 0.006495), Leukocyte transendothelial migration (*p* = 0.008752), and Chemical carcinoma-reactive oxygen species (*p* = 0.009001), and expressed in signaling pathways, such as Chemokine signaling pathway (*p* = 0.033944) and PI3K-Akt signaling pathway (*p* = 0.042931), and some cellular life processes, Examples include Osteoclast differentiation (*p* = 0.001493), Phagosome (*p* = 0.002308), and Cell cycle(*p* = 0.002596).

#### Construction of PPI network and screening of key genes

**Fig. 8:**
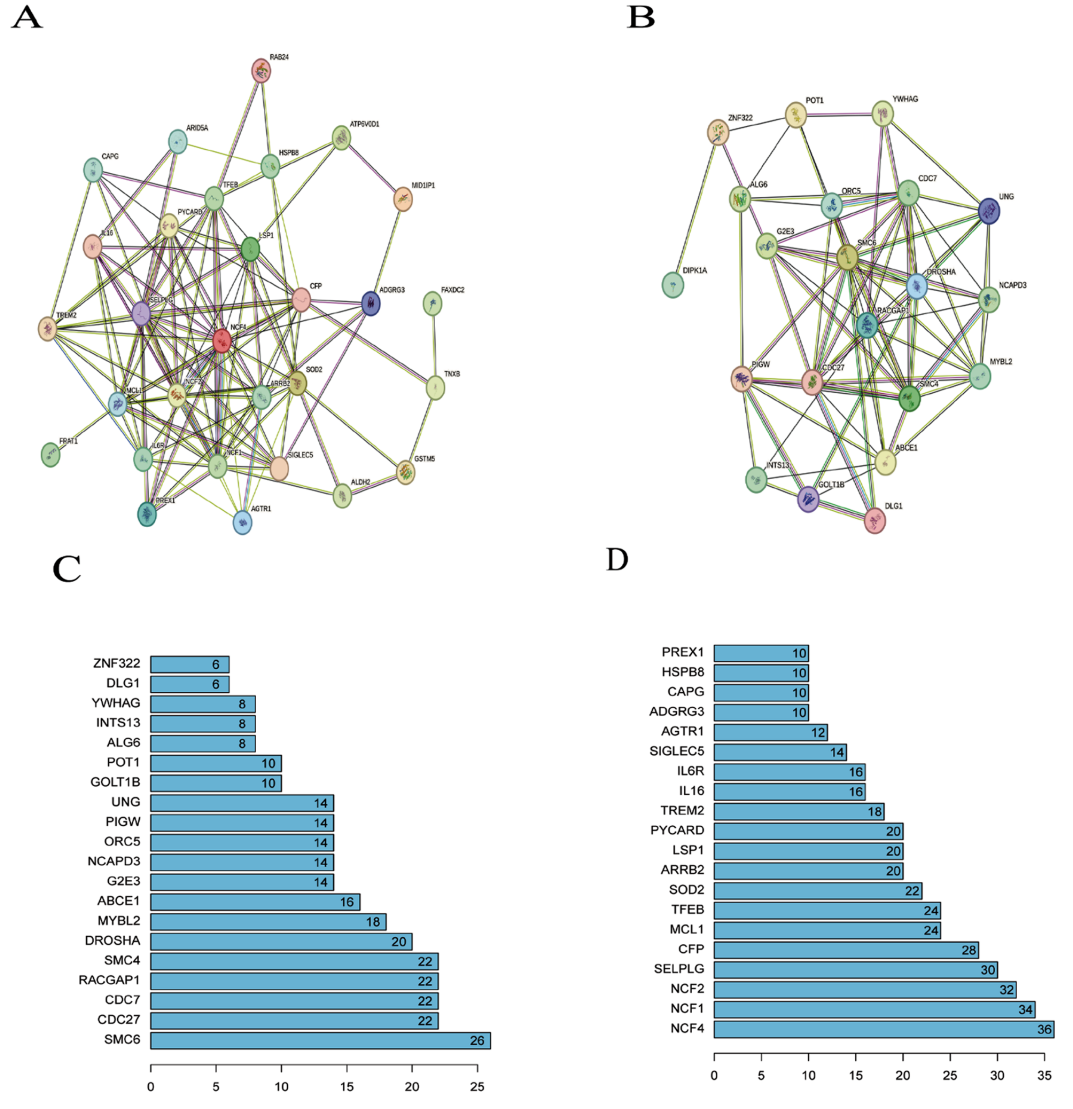
Protein-protein interaction (PPI) analysis of (**A**) upregulated co-DEGs; (**B**) downregulated co-DEGs; (**C**) hub genes in downregulated co-DEGs; (**D**) hub genes in upregulated co-DEGs

In this study, the STRING database was used to construct the PPI network of up-regulated and down-regulated co-DEGs to screen the hub genes of co-DEGs further. The PPI network of co-DEGs that were both up-regulated consisted of 21 genes and 296 edges, and the PPI network of co-DEGs that were down-regulated consisted of 29 genes and 448 edges (Fig. 8A-B). Key hub genes were screened using the cytoHubba plugin in Cytoscape software. According to the visualization results of the PPI network combined with the key nodes of the PPI network, the hub key genes of 10 co-DEGs were finally screened out in this study, which were *SMC6, CDC27, CDC7, RACGAP1, SMC4, NCF4, NCF1, NCF2, SELPLG* and *CFP* (Fig. 8C-D).

### Receiver operating characteristic (ROC) curve

**Fig. 9:**
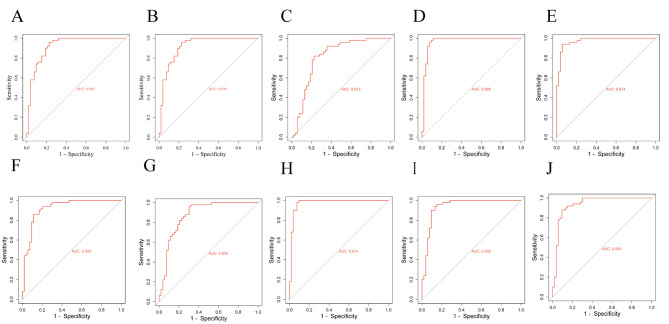
ROC curve of co-DEGs in NSCLC. (**A**) SMC6; (**B**) CDC27; (**C**) CDC7; (**D**) RACGAP1; (**E**) SMC4; (**F**) NCF4; (**G**) NCF1; (**H**) NCF2; (**I**) SELPLG; (**J**) CFP

**Fig. 10:**
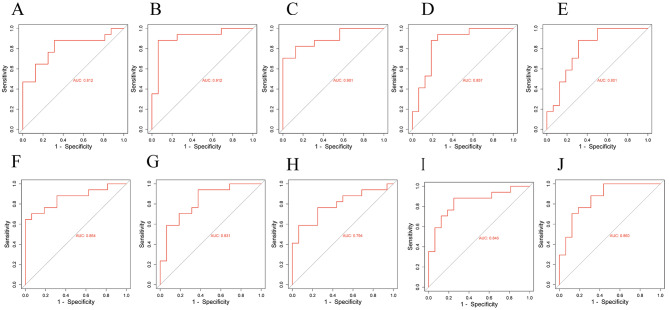
ROC curve of co-DEGs in T2DM. (**A**) SMC6; (**B**) CDC27; (**C**) CDC7; (**D**) RACGAP1; (**E**) SMC4; (**F**) NCF4; (**G**) NCF1; (**H**) NCF2; (**I**) SELPLG; (**J**) CFP

ROC curve was used to verify the diagnostic value of hub genes in NSCLC and T2DM. For patients with NSCLC, the AUCs of *SMC6, CDC27, CDC7, RACGAP1, SMC4, NCF4, NCF1, NCF2, SELPLG*, and *CFP* were 0.920, 0.920, 0.812, 0.968, 0.974, 0.920, 0.875, 0.974, 0.939, and 0.935, respectively (Fig. 9A-J). For patients with *T2DM*, the AUCs of *SMC6, CDC27, CDC7, RACGAP1, SMC4, NCF4, NCF1, NCF2, SELPLG*, and *CFP* were 0.812, 0.912, 0.901, 0.857, 0.801, 0.864, 0.831, 0.794, 0.846, and 0.860, respectively (Fig. 10A-J).

The hub-genes had good diagnostic efficiency and high diagnostic value in both NSCLC and T2DM (0.9 > AUC > 0.7). In addition, among the up-regulated genes, *RACGAP1* had a high diagnostic value for NSCLC and T2DM. *SMC4* had a high diagnostic value for NSCLC and T2DM among the down-regulated genes.

### Evaluation of immune cell infiltration

**Fig. 11:**
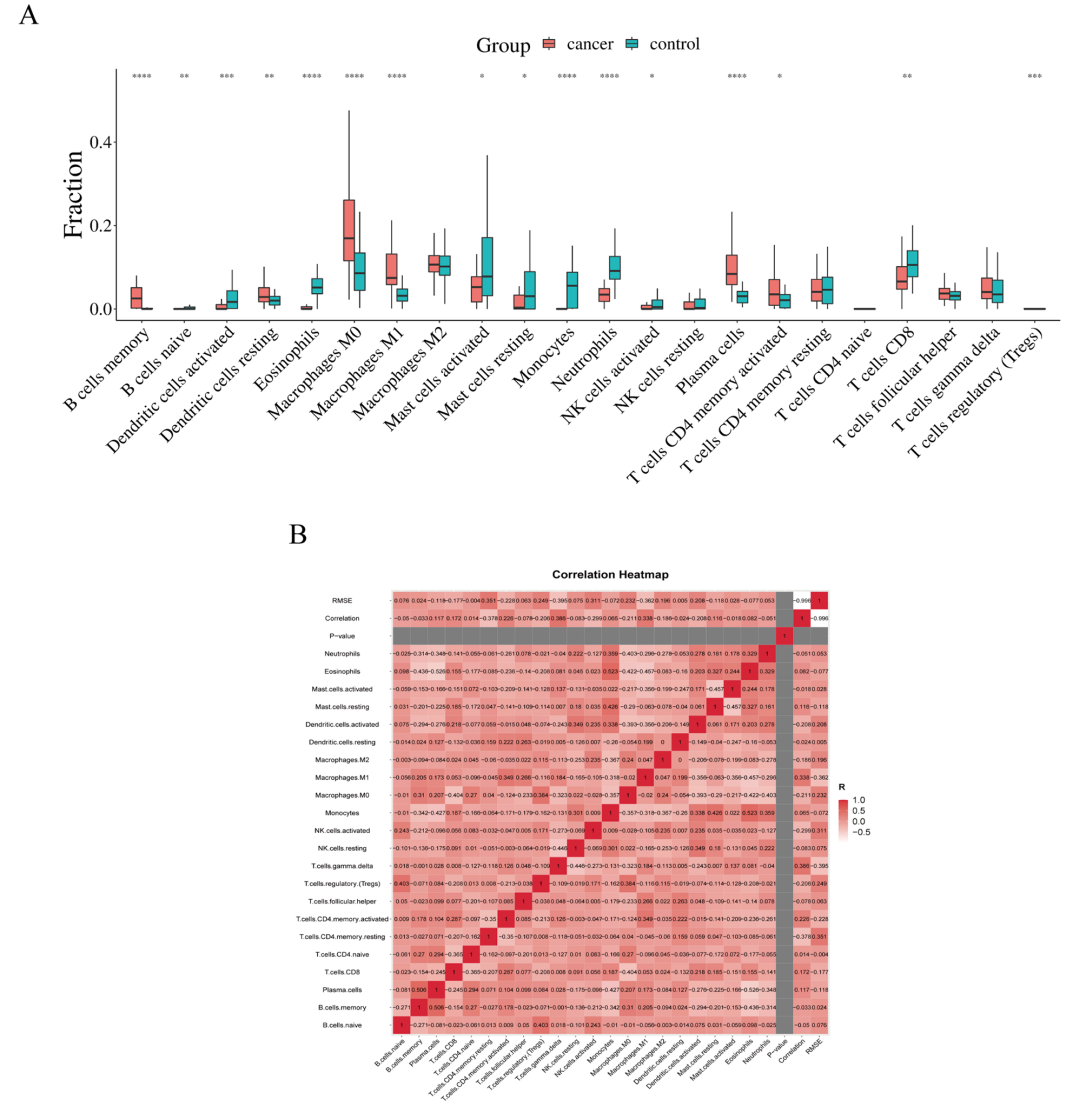
(**A**) Analysis of differences in immune cells between the lung cancer and the control group. The horizontal axis represents the different immune cells, the vertical axis represents the proportion of immune cells; (**B**) Immune cell proportion correlation matrix. *p < 0.05;**p < 0.01;***p < 0.001;****p < 0.0001

Box plots of differences in immune cell infiltration showed that compared with the control group, memory B cells, activated myeloid dendritic cells, M0 and M1 macrophages, plasma cells, CD4 + memory activated T cells were significantly increased in the lung cancer group. However, resting myeloid dendritic cells, eosinophils, activated mast cells, Monocytes, neutrophils, and CD8 + T cells were significantly reduced in the lung cancer group (Fig. 11A). The corrplot package in R software was used for the correlation analysis of immune cells. As shown in Fig. 11B, the numbers in the squares represent the correlation coefficients between the corresponding immune cells. The combinations with high positive correlation include memory B and plasma cells, Eosinophils and Monocytes. The combinations with high negative correlation were Eosinophils and plasma cells.

**Fig. 12:**
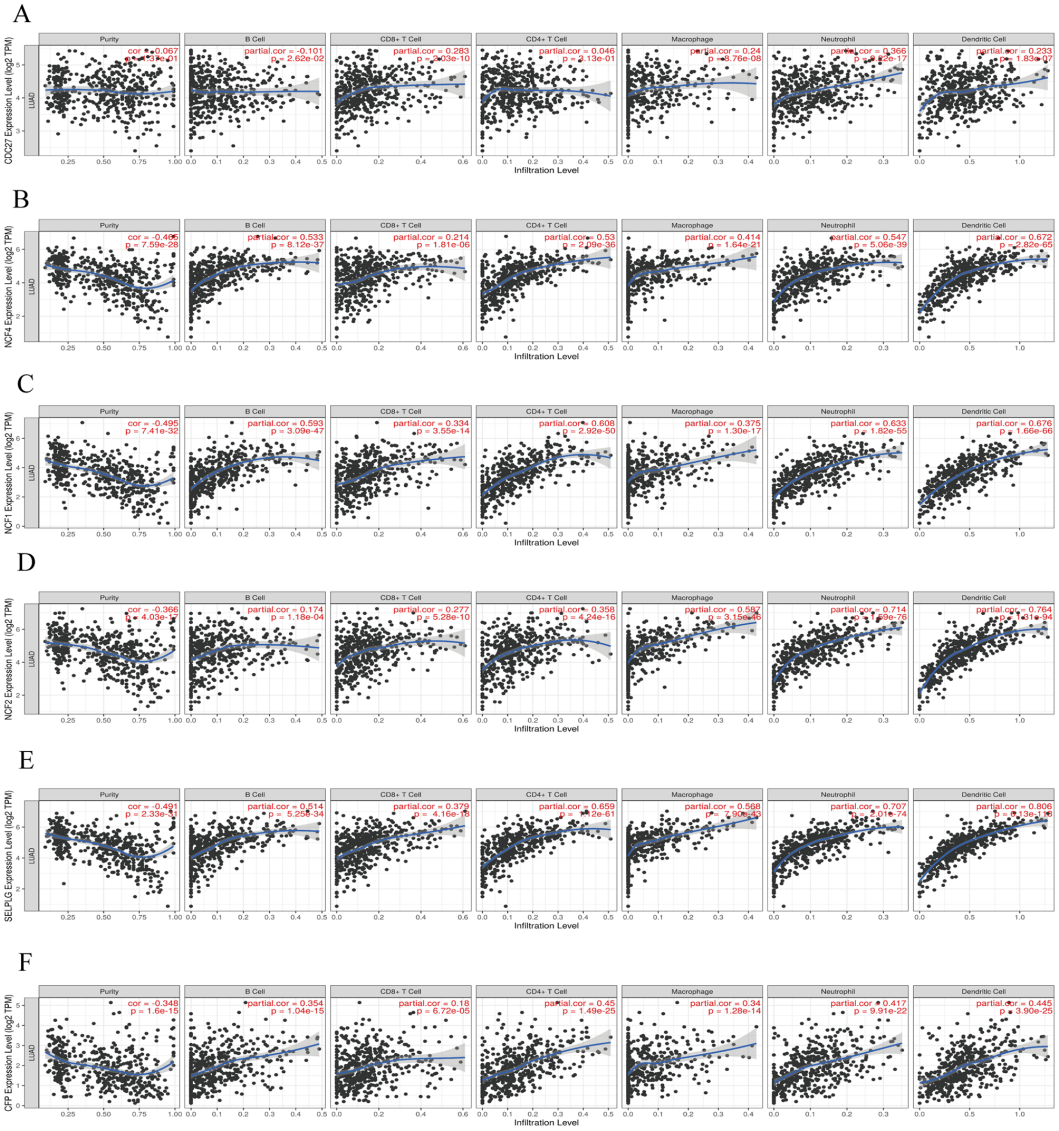
Correlation between hub genes and immune cell components in NSCLC and T2DM. (**A**) *CDC27;* (**B**) *NCF4;* (**C**) *NCF1;* (**D**) *NCF2;* (**E**) *SELPLG;* (**F**) *CFP*

Then, we further explored the spear-man correlation coefficient between hub genes and the degree of infiltration of immune cells. As a result, all hub genes were associated with immune cells. Using correlation scatter plots, we visualized the six hub genes most strongly associated with immune cells (Fig. 12). NCF1, 2, 4 and SELPLG genes were positively correlated with B cells, CD4 + T cells, macrophages, neutrophils, and dendritic cells. The CFP gene was positively correlated with CD4 + T cells, neutrophils, and dendritic cells.

## Discussion

In clinical work, we have observed an increasing number of patients with pulmonary nodules or lung cancer who also have diabetes or hyperglycemia. In addition to treating the symptoms to lower blood glucose, clinicians need to investigate the potential relationship and interaction between these two diseases during clinical diagnosis and treatment. T2DM is a risk factor for various malignancies due to reduced pancreatic insulin secretion and insulin resistance, and it is also a known factor in tumor growth [23]. Some basic research has indicated that factors such as hyperglycemia, hyperinsulinemia, glycosylation, inflammation, and hypoxia may be potential mechanisms for promoting the proliferation and invasiveness of lung tumor cells [24].

Hyperglycemia is not only an indicator of prediabetes/diabetes but also one of a critical factors affecting cancer treatment. The molecular mechanisms underlying lung cancer in T2DM remain unclear. Therefore, exploring the co-expressed gene levels of lung cancer patients and diabetes is crucial for personalized treatment and improving prognosis [25]. This is the first time to examine the common genes and characteristics of NSCLC and T2DM using bioinformatics methods such as WGCNA and immune infiltration analysis, which can guide early detection, better treatment and timely prevention.

Although these studies suggest a potential link between diabetes and the development of lung cancer, previous large cohort studies [26, 27] have produced conflicting results regarding the relationship between diabetes and lung cancer risk, showing both reduced or increased risk and no association. These studies included relatively heterogeneous populations, and most lacked the power to assess the association between diabetes and lung cancer risk.

Therefore, we conducted a comprehensive statistical analysis using the NHANES 2000–2018 dataset to investigate the association between various indicators of diabetes and the risk of lung cancer. After accounting for all confounding factors, we discovered that prediabetes and type 2 diabetes were still significantly linked to an increased risk of lung cancer. Both fasting plasma glucose and glycosylated haemoglobin had an inverted U-shaped association with the risk of lung cancer. Therefore, we hypothesize that hyperglycemia or diabetes is correlated with an elevated risk of lung cancer, although the specific genetic mechanisms need to be further studied.

Based on the results of the epidemiological analysis, we further explored the potential molecular mechanisms related to NSCLC and T2DM using transcriptome data. This study analyzed GSE18842, GSE118370, GSE26168 and GSE15932 datasets from the GEO database. First, WGCNA analysis explored the core modules significantly related to the disease. Limma analysis identified 57 co-expressed genes, 25 up-regulated co-expressed genes, and 32 down-regulated co-expressed genes. Then, KEGG pathway enrichment analysis and PPI network were constructed to identify hub key genes in co-DEGs. KEGG pathway analysis of co-DEGs mainly focused on several inflammatory and metabolic diseases and signaling pathways. These co-expressed genes were related to tumor, immunity and inflammation. Based on the above analysis, 10 hub genes were identified in this study, namely *SMC6, CDC27, CDC7, RACGAP1, SMC4, NCF4, NCF1, NCF2, SELPLG* and *CFP*. ROC curve analysis showed these 10 hub genes had good diagnostic values for NSCLC and T2DM (0.9 > AUC > 0.8). Among them, *RACGAP1* and *SMC4* had the best predictive value. Through bioinformatics analysis, the co-expressed genes of NSCLC and T2DM were obtained in this study, which provides a basis for the early diagnosis and treatment of the diseases.

Six genes of *SMC* family members form *SMC* protein complexes by creating heterodimers and binding to other proteins in the cell, including adhesin (*SMC*1/3), clustin (*SMC*2/4) and *SMC*5/6 complex [28, 29]. The *SMC* complex regulates genome stability and homeostasis in the nucleus by controlling chromatid condensation, DNA replication and repair, and homologous chromosome pairing in meiosis [30].

Previous studies [31] have demonstrated that the protein products of *SMC* genes can act not only as transcription factors that promote carcinogenesis but also as tumor suppressors based on their aberrant expression patterns in some organs. An increasing number of studies [32] have shown that *SMC4* also plays essential roles in the non-mitotic phase of the cell cycle, such as maintaining the silent state of gene expression, heterochromatin organization and DNA repair. Recent studies have shown that *SMC4* is aberrantly expressed in HCC and colon cancer. However, the underlying mechanism of *SMC4* in carcinogenesis has not been investigated. There is no research on *SMC4* and *SMC6* in lung cancer and diabetes, which needs further exploration.

*CDC27* comprises 33 specific exons and can produce multiple transcripts through alternative splicing, resulting in 22 different mRNAs, 13 of which can be translated into functional proteins. The primary function of *CDC27* is encoded by 19 exons, with 830 and 824 amino acids, respectively [33]. *APC/C* can regulate cell division, genome stability, cell differentiation, carcinogenesis, autophagy, cell death, and energy metabolism. Most of these functions, which are essential in tumor pathogenesis, may be regulated by *CDC27* in *APC/C*. Changes in *CDC27* at DNA, RNA, and protein levels, and post-translational modifications could impact cell division [34]. The discovery of its abnormal expression in various malignant tumors provides a new direction for the early diagnosis, treatment evaluation and prognosis of these malignant tumors [35]. *CDC27* has not been investigated in diabetes thus far, and we need to verify further its potential as a common target in lung cancer and diabetes.

Cell division cycle 7 (*CDC7*) is a conserved serine-threonine kinase essential for initiating DNA replication [36]. *CDC7* controls S-phase checkpoints in the DNA damage response (DDR) by reducing checkpoint signaling and triggering DNA replication reinitiation. *CDC7* may also phosphorylate claspin and activate the *ATR-CHK1* checkpoint pathway [37]. *CDC7* expression is minimal or undetectable in normal tissues and cell lines but highly expressed in many human cancer and tumor cell lines. Silencing *CDC7* in cancer cells impairs S-phase progression and induces p53-independent apoptosis but does not affect normal cells [38]. Thus, *CDC7* becomes an attractive target for cancer therapy.

*RACGAP1* is a component of centrospirin that is crucial for the activation of cell division and is believed to be a member of the Rho GTpase-activating protein family [39]. *RACGAP1* binds to GTP-bound Rac1 and serves as both a mediator of tyrosine phosphorylation of the signal transducer and activator of transcription (STAT) protein family and as a nuclear chaperone of phosphorylated STATs containing nuclear localization signals [40]. Racgap1 has multiple functions, including anti-apoptosis, proliferation, differentiation, and inflammation [41].

*RACGAP1* also controls the activity of Rho proteins, including Rac and *CDC42*, to influence cell shape, migration, and polarization. *RACGAP1* has been shown to play essential roles in multiple cancers through its high expression. *RACGAP1* can increase the potential for malignancy and can be used as a biomarker for lymph node metastasis and prognosis in colorectal cancer [42]. *RACGAP1* has also been shown to drive breast cancer metastasis by regulating ECT2-dependent mitochondrial quality control [43]. Liang et al. ‘s study [44] was the first to link *RACGAP1* to lung cancer, observing that downregulation of *RACGAP1* in cultured lung cancer cells by RNA interference led to defects in cell division. Rac1 is a master regulator of cytoskeleton remodelling and is vital for insulin particle fusion and transport and subsequent secretion in pancreatic β-cells. *APPL2* interacts with *RacGAP1*, inhibiting the conversion of active GTP-bound Rac1 to inactive GDP-bound Rac1 [45]. The *APPL2-RacGAP-Rac1* signaling axis is essential for tightly regulating *GSIS* and subsequent glucose homeostasis.

In the last decade, with the rapid development of tumor immunotherapy, immune-infiltrating cells in the tumor environment have garnered increasing attention. Many of their functions have been identified as targets for treating malignant melanoma and lung cancer [46]. It is crucial to investigate the correlation between elevated gene expression in tumor tissues and tumor infiltration by different types of immune cells. In this study, the CIBERSOTR algorithm was used to evaluate the types of immune cells infiltration in the lung cancer group. It was discovered that various immune cell subtypes were closely related to critical biological processes of lung cancer.

Memory B cells, activated myeloid dendritic cells, M0 and M1 macrophages, plasma cells, and CD4 + memory activated T cells showed significant increases in the lung cancer group, while resting myeloid dendritic cells, eosinophils, activated mast cells, Monocytes, neutrophils, and CD8 + T cells exhibited significant decreases. In addition, we delved into the correlation between hub genes and immune cells. All Hub genes were found to be significantly correlated with immune cells. Notably, NCF1, 2, 4 and SELPLG genes were positively correlated with B cells, CD4 + T cells, macrophages, neutrophils and dendritic cells.

Neutrophil cytoplasmic factor 1 (*NCF1*), neutrophil cytoplasmic factor 2 (*NCF2*), and neutrophil cytoplasmic factor 4 (*NCF4*) are also known as p47phox, p67phox, and p40phox, respectively [47]. They belong to the NADPH oxidase complex, a cytoplasmic component whose polymorphism is a significant factor associated with autoimmune diseases and is most likely caused by the modulation of peroxides. Increasing evidence suggests that *NCF1, NCF2* and *NCF4* play essential roles in tumorigenesis and progression [48].

The selection P-ligand gene (*SELPLG*), also known as *CD162* and *PSGL-1*, is expressed in bone marrow cells and stimulated T lymphocytes [49]. This protein plays a crucial role in leukocyte trafficking during inflammation by binding leukocytes to activated platelets or selectin-expressing endothelial cells. Recently, *it has been shown that SELPLG* acts as an immune checkpoint regulator in colorectal cancer [50], head and neck squamous cell carcinoma, and melanoma [51], making it a potential novel therapeutic target for cancer.

In the analysis, we found a significant positive correlation between the CFP gene and CD4 + T cells, neutrophils and dendritic cells. *CFP*, which encodes a plasma glycoprotein, binds and stabilizes the labile C3 convertase (*C3bBb*) in the complement system and actively regulates the innate immune system in the alternative pathway (AP). *CFP* is mainly synthesized and secreted by leukocytes. Furthermore, various immune cells, particularly mature neutrophils, significantly affect serum CFP expression levels [52]. Research has indicated that *CFP* may serve as an independent risk factor for the prognosis of lung cancer and could be involved in regulating relevant immune mechanisms in the tumor microenvironment [53]. However, there is limited research on the relationship between *CFP* and tumors, and the specific mechanism needs further study.

Diabetes is a metabolic disease that arises from inflammation in a complex immune process. Insulin resistance, caused by the inhibition of insulin signaling, triggers a series of immune responses that exacerbate the inflammatory state, leading to hyperglycemia. Crosstalk between pathogenic CD4 + and CD8 + T cells and CD11c + M1 macrophages in obese adipose tissue further enhances the inflammatory immune response induced by adipocyte apoptosis and macrophage infiltration, exacerbating adipose tissue inflammation and peripheral insulin resistance. In addition, neutrophil dysfunction, macrophage dysfunction, cytokine and complement production are all related to the development of T2DM. Our findings suggest that the co-expressed genes of lung cancer and diabetes may mediate the interaction between lung cancer and diabetes through the immune cell infiltration pathway, which needs further experimental verification.

Our findings indicated the co-expression of genes in NSCLC and T2DM. We have established a genetic basis for the common pathogenesis of NSCLC and T2DM. Additionally, the identified hub genes may provide new insights for the early diagnosis of future diseases. Identifying co-expressed genes in both conditions may alert clinicians to the potential development of lung cancer in patients with lung nodules expressing these hub genes, allowing for early clinical intervention. Genetic testing is currently widely used for the early diagnosis of diseases. The co-expressed genes of NSCLC and T2DM that we have proposed can, to some extent, distinguish between NSCLC and non-NSCLC subjects and between type 2 diabetes and non-type 2 diabetes subjects. However, it is necessary to clarify further whether there are expression differences of these hub genes in NSCLC and T2DM, and whether there are cumulative effects of gene expression between the diseases, which requires further experimental validation. Additionally, we can also use co-expressed gene targets to predict personalized treatment for lung cancer combined with T2DM. Before clinical use, these drugs require extensive animal research, clinical trials, adverse reaction monitoring, and approval from the Food and Drug Administration.

In summary, *SMC6, CDC27, CDC7, RACGAP1, SMC4, NCF1,2,4, SELPLG* and *CFP* are significantly associated with T2DM and lung cancer, making them potential target genes for disease prediction and treatment in the future. Further experiments can be designed around these genes to verify the pathogenic mechanism and achieve early disease prediction. However, our study has some limitations. Firstly, we used the NHANES 2000–2018 dataset for the epidemiologic analysis, which may limit the extrapolation of statistical results as all participants were from the United States. At the same time, although we have included extensive sample data spanning 18 years, the number of people with lung cancer in this dataset is small, and more large sample epidemiological investigations are needed in the future. Secondly, we have only reached a preliminary conclusion that the bioinformatics dataset from GEO needs to be validated by our independent experiments. Further validation in cell and animal models needs to be performed. Future studies will focus on verifying the clinical application of these genes in individualized cancer therapy.

## Conclusion

Based on the NHANES database, we found that diabetes status and glycemic measures (fasting plasma glucose, glycated haemoglobin) were significantly associated with an increased risk of lung cancer. We then used bioinformatics methods to screen and validate the co-expressed genes between lung cancer and T2DM. We identified the critical pathogenic genes *SMC6, CDC27, CDC7, RACGAP1, SMC4, NCF1,2,4*, and *SELPLG*, which exhibited good predictive value. Furthermore, these core genes were associated with dysregulated immune cells. Together, these dysregulated core genes and immune cells offer potential research avenues for lung cancer with diabetes. It is anticipated that early genetic screening and individualized treatment for diabetic patients with high-risk factors of lung cancer will be conducted. In the future, more basic experiments are required to explore the potential mechanisms by which co-expressed genes regulate diseases.

### Electronic supplementary material

Below is the link to the electronic supplementary material.

Supplementary Material 1: Table S1. Details of GEO datasets.

## Data Availability

Data and material are available on reasonable request from the corresponding author.

## Abbreviations

FPG: fasting plasma glucose
Fins: fasting insulins
BMI: body mass index
NHANES: National Health and Nutrition Examination Survey
T2DM: Type 2 diabetes mellitus
GEO: Gene expression omnibus
PPI: Protein-protein interaction
TC: Total cholesterol
TG: Triglyceride
LDL: Lower density lipoprotein
HDL: Higher density lipoprotein
WGCNA: Weighted gene co-expression network analysis
DEGs: Differentially expressed genes
KEGG: Kyoto Encyclopedia of Genes and Genomes
GO: Gene Ontology
BP: Biological processes
CC: Cellular component
MF: Molecular function
NCF: Neutrophil cytoplasmic factor
SELPLG: The selection P-ligand gene
CDC: Cell division cycle
(PSGL-1): P-selectin glycoprotein ligand-1

## References

[1] Bray F, Ferlay J, Soerjomataram I, Siegel RL, Torre LA, Jemal A (2018). Global cancer statistics 2018: GLOBOCAN estimates of incidence and mortality worldwide for 36 cancers in 185 countries. CA Cancer J Clin.

[2] de Koning HJ, van der Aalst CM, de Jong PA, Scholten ET, Nackaerts K, Heuvelmans MA (2020). Reduced lung-Cancer mortality with volume CT screening in a Randomized Trial. N Engl J Med.

[3] Leiter A, Charokopos A, Bailey S, Gallagher EJ, Hirsch FR, LeRoith D (2021). Assessing the association of diabetes with lung cancer risk. Transl Lung Cancer Res.

[4] Tomkins M, Lawless S, Martin-Grace J, Sherlock M, Thompson CJ (2022). Diagnosis and management of Central Diabetes Insipidus in adults. J Clin Endocrinol Metab.

[5] Màrmol JM, Carlsson M, Raun SH, Grand MK, Sørensen J, Lang Lehrskov L (2023). Insulin resistance in patients with cancer: a systematic review and meta-analysis. Acta Oncol.

[6] Sylow L, Vind BF, Kruse R, Møller PM, Wojtaszewski JFP, Richter EA, et al. Circulating Follistatin and activin A and their regulation by insulin in obesity and type 2 diabetes. J Clin Endocrinol Metab. 2020;105(5). 10.1210/clinem/dgaa090. 10.1210/clinem/dgaa090 32112102

[7] Kim DS, Scherer PE (2021). Obesity, diabetes, and increased Cancer progression. Diabetes Metab J.

[8] Luo J, Hendryx M, Qi L, Ho GY, Margolis KL (2016). Pre-existing diabetes and lung cancer prognosis. Br J Cancer.

[9] Tseng CH (2014). Diabetes but not insulin increases the risk of lung cancer: a Taiwanese population-based study. PLoS ONE.

[10] Yang J, Yang J (2019). Hyperglycemic ADR distribution of Doxorubicin from VigiBase. Am J Ther.

[11] Yang J, Jia B, Qiao Y, Chen W, Qi X (2016). Variations of blood glucose in cancer patients during chemotherapy. Niger J Clin Pract.

[12] Ding J, Tang J, Chen X, Men HT, Luo WX, Du Y (2013). Expression characteristics of proteins of the insulin-like growth factor axis in non-small cell lung cancer patients with preexisting type 2 diabetes mellitus. Asian Pac J Cancer Prev.

[13] Yu H, Spitz MR, Mistry J, Gu J, Hong WK, Wu X (1999). Plasma levels of insulin-like growth factor-I and lung cancer risk: a case-control analysis. J Natl Cancer Inst.

[14] Meng X, Li Z, Zhou S, Xiao S, Yu P (2019). miR-194 suppresses high glucose-induced non-small cell lung cancer cell progression by targeting NFAT5. Thorac Cancer.

[15] Nakazawa K, Kurishima K, Tamura T, Ishikawa H, Satoh H, Hizawa N (2013). Survival difference in NSCLC and SCLC patients with diabetes mellitus according to the first-line therapy. Med Oncol.

[16] Hartwell ML, Khojasteh J, Wetherill MS, Croff JM, Wheeler D (2019). Using Structural equation modeling to examine the influence of social, behavioral, and Nutritional Variables on Health outcomes based on NHANES Data: addressing Complex Design, Nonnormally distributed variables, and missing information. Curr Dev Nutr.

[17] Yi ZH, Luther Y, Xiong GH, Ni YL, Yun F, Chen J (2020). Association between diabetes mellitus and lung cancer: Meta-analysis. Eur J Clin Invest.

[18] Barrett T, Wilhite SE, Ledoux P, Evangelista C, Kim IF, Tomashevsky M (2013). NCBI GEO: archive for functional genomics data sets–update. Nucleic Acids Res.

[19] Xia Y, Xia C, Wu L, Li Z, Li H, Zhang J. Systemic Immune inflammation index (SII), system inflammation response index (SIRI) and risk of all-cause Mortality and Cardiovascular Mortality: a 20-Year Follow-Up Cohort Study of 42,875 US adults. J Clin Med. 2023;12(3). 10.3390/jcm12031128. 10.3390/jcm12031128 PMC9918056 36769776

[20] Weng L, Xu Z, Chen Y, Chen C (2023). Associations between the muscle quality index and adult lung functions from NHANES 2011–2012. Front Public Health.

[21] 2 (2020). Classification and diagnosis of diabetes: standards of Medical Care in Diabetes-2020. Diabetes Care.

[22] Newman AM, Liu CL, Green MR, Gentles AJ, Feng W, Xu Y (2015). Robust enumeration of cell subsets from tissue expression profiles. Nat Methods.

[23] Liao YF, Yin S, Chen ZQ, Li F, Zhao B (2018). High glucose promotes tumor cell proliferation and migration in lung adenocarcinoma via the RAGE–NOXs pathway. Mol Med Rep.

[24] Kang X, Kong F, Wu X, Ren Y, Wu S, Wu K (2015). High glucose promotes tumor invasion and increases metastasis-associated protein expression in human lung epithelial cells by upregulating heme oxygenase-1 via reactive oxygen species or the TGF-β1/PI3K/Akt signaling pathway. Cell Physiol Biochem.

[25] Jee SH, Ohrr H, Sull JW, Yun JE, Ji M, Samet JM (2005). Fasting serum glucose level and cancer risk in Korean men and women. JAMA.

[26] Hu Y, Zhang X, Ma Y, Yuan C, Wang M, Wu K (2021). Incident Type 2 diabetes duration and Cancer risk: a prospective study in two US cohorts. J Natl Cancer Inst.

[27] Dankner R, Boffetta P, Balicer RD, Boker LK, Sadeh M, Berlin A (2016). Time-Dependent risk of Cancer after a diabetes diagnosis in a cohort of 2.3 million adults. Am J Epidemiol.

[28] Park HJ, Joh HK, Choi S, Park SM (2019). Type 2 diabetes mellitus does not increase the risk of lung cancer among never-smokers: a nationwide cohort study. Transl Lung Cancer Res.

[29] Chiotaki R, Polioudaki H, Theodoropoulos PA (2014). Differential nuclear shape dynamics of invasive andnon-invasive breast cancer cells are associated with actin cytoskeleton organization and stability. Biochem Cell Biol.

[30] Hanahan D, Weinberg RA (2011). Hallmarks of cancer: the next generation. Cell.

[31] Wang D, Wang L, Zhang Y, Zhao Y, Chen G (2018). Hydrogen gas inhibits lung cancer progression through targeting SMC3. Biomed Pharmacother.

[32] Feng Y, Liu H, Duan B, Liu Z, Abbruzzese J, Walsh KM (2019). Potential functional variants in SMC2 and TP53 in the AURORA pathway genes and risk of pancreatic cancer. Carcinogenesis.

[33] Kazemi-Sefat GE, Keramatipour M, Talebi S, Kavousi K, Sajed R, Kazemi-Sefat NA (2021). The importance of CDC27 in cancer: molecular pathology and clinical aspects. Cancer Cell Int.

[34] Qiu L, Zhou R, Luo Z, Wu J, Jiang H (2022). CDC27-ODC1 Axis promotes metastasis, accelerates ferroptosis and predicts poor prognosis in Neuroblastoma. Front Oncol.

[35] Chang CL, Huang KC, Chen TW, Chen WT, Huang HH, Liu YL (2022). Prognostic and clinical significance of subcellular CDC27 for patients with rectal adenocarcinoma treated with adjuvant chemotherapy. Oncol Lett.

[36] Hughes S, Elustondo F, Di Fonzo A, Leroux FG, Wong AC, Snijders AP (2012). Crystal structure of human CDC7 kinase in complex with its activator DBF4. Nat Struct Mol Biol.

[37] Montagnoli A, Bosotti R, Villa F, Rialland M, Brotherton D, Mercurio C (2002). Drf1, a novel regulatory subunit for human Cdc7 kinase. Embo j.

[38] Cao JX, Lu Y, Qi JJ, An GS, Mao ZB, Jia HT (2014). MiR-630 inhibits proliferation by targeting CDC7 kinase, but maintains the apoptotic balance by targeting multiple modulators in human lung cancer A549 cells. Cell Death Dis.

[39] Lee JS, Kamijo K, Ohara N, Kitamura T, Miki T (2004). MgcRacGAP regulates cortical activity through RhoA during cytokinesis. Exp Cell Res.

[40] Zhao WM, Fang G (2005). MgcRacGAP controls the assembly of the contractile ring and the initiation of cytokinesis. Proc Natl Acad Sci U S A.

[41] Jantsch-Plunger V, Gönczy P, Romano A, Schnabel H, Hamill D, Schnabel R (2000). CYK-4: a rho family gtpase activating protein (GAP) required for central spindle formation and cytokinesis. J Cell Biol.

[42] Ren K, Zhou D, Wang M, Li E, Hou C, Su Y (2021). RACGAP1 modulates ECT2-Dependent mitochondrial quality control to drive breast cancer metastasis. Exp Cell Res.

[43] Yin C, Toiyama Y, Okugawa Y, Shigemori T, Yamamoto A, Ide S (2019). Rac GTPase-Activating protein 1 (RACGAP1) as an oncogenic enhancer in esophageal carcinoma. Oncology.

[44] Liang Y, Liu M, Wang P, Ding X, Cao Y (2013). Analysis of 20 genes at chromosome band 12q13: RACGAP1 and MCRS1 overexpression in nonsmall-cell lung cancer. Genes Chromosomes Cancer.

[45] Wang B, Lin H, Li X, Lu W, Kim JB, Xu A (2020). The adaptor protein APPL2 controls glucose-stimulated insulin secretion via F-actin remodeling in pancreatic β-cells. Proc Natl Acad Sci U S A.

[46] Tinoco R, Otero DC, Takahashi AA, Bradley LM (2017). PSGL-1: a New Player in the Immune Checkpoint Landscape. Trends Immunol.

[47] Chen Y, He F, Wang R, Yao M, Li Y, Guo D (2021). NCF1/2/4 are prognostic biomarkers related to the Immune infiltration of kidney renal clear cell carcinoma. Biomed Res Int.

[48] Li Q, Zhong J, Luo H, Urbonaviciute V, Xu Z, He C (2022). Two major genes associated with autoimmune arthritis, Ncf1 and Fcgr2b, additively protect mice by strengthening T cell tolerance. Cell Mol Life Sci.

[49] Molnár Z, Bánlaki Z, Somogyi A, Herold Z, Herold M, Guttman A (2020). Diabetes-specific modulation of Peripheral Blood Gene expression signatures in Colorectal Cancer. Curr Mol Med.

[50] Sanati N, Iancu OD, Wu G, Jacobs JE, McWeeney SK (2018). Network-based predictors of progression in Head and Neck squamous cell carcinoma. Front Genet.

[51] Sun S, Shi R, Xu L, Sun F (2022). Identification of heterogeneity and prognostic key genes associated with uveal melanoma using single-cell RNA-sequencing technology. Melanoma Res.

[52] Cui G, Geng L, Zhu L, Lin Z, Liu X, Miao Z (2021). CFP is a prognostic biomarker and correlated with immune infiltrates in gastric Cancer and Lung Cancer. J Cancer.

[53] Block I, Müller C, Sdogati D, Pedersen H, List M, Jaskot AM (2019). CFP suppresses breast cancer cell growth by TES-mediated upregulation of the transcription factor DDIT3. Oncogene.

