# Supplementary material for: Epidemiological and transcriptome data identify shared gene signatures and immune cell infiltration in type 2 diabetes and non-small cell lung cancer
Source: Diabetol Metab Syndr. 2024 Mar 12;16:64. doi: 10.1186/s13098-024-01278-z (PMC10929160; doi:10.1186/s13098-024-01278-z)
Supplement: Supplementary file 1 — Supplementary Material 1: Table S1. Details of GEO datasets. [file 13098_2024_1278_MOESM1_ESM.docx]

TableS1. Datasets details

| Disease | GEO ID | Platform | Organism | Type | Samples  (Control vs.Case  ) | Race | PMID |
| --- | --- | --- | --- | --- | --- | --- | --- |
| NSCLC | GSE18842 | GPL570 | Homo sapiens | Lung tissue | 44vs47 | Caucasian | 20878980 |
|  | GSE118370 | GPL570 | Homo sapiens | Homo sapiens | 6vs6 | Chinese | 30545439 |
| Validation | GSE135304 | GPL10588 | Homo sapiens | Blood | 200vs200 | Caucasian | 30487137 |
| T2DM | GSE26168 | GPL6883 | Homo sapiens | Blood | 18vs18 | Singaporean | 33149760 |
|  | GSE15932 | GPL570 | Homo sapiens | Blood | 8vs8 | Chinese | 34257566 |
| Validation | GSE7014 | GPL570 | Homo sapiens | Skeletal Muscle | 6vs20 | Caucasian | 31797865 |
